# Supplementary figures and images for: Deep mutagenesis scanning using whole trimeric SARS-CoV-2 spike highlights the importance of NTD-RBD interactions in determining spike phenotype
Source: PLoS Pathog. 2023 Aug 3;19(8):e1011545. doi: 10.1371/journal.ppat.1011545 (PMC10426949; doi:10.1371/journal.ppat.1011545)

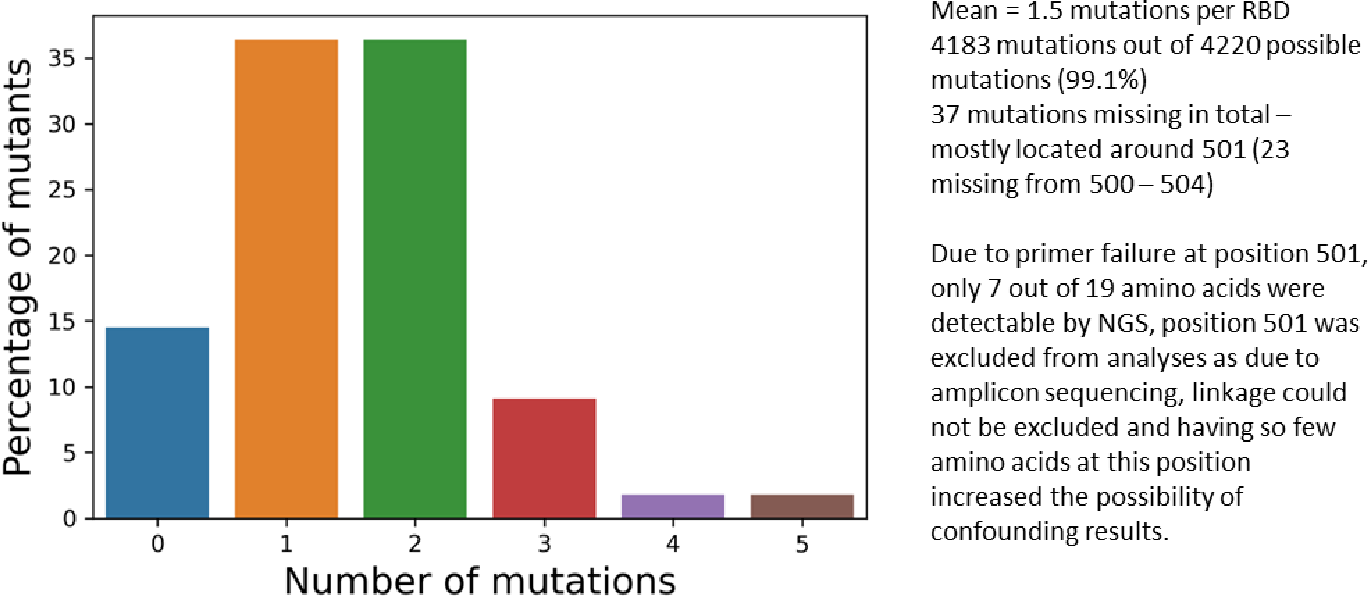

Supplement: S1 Fig — Distribution of number of mutations per RBD. Following transformation with plasmid containing whole Alpha spike with the RBD library, 50 colonies, were picked, mini prepped and Sanger sequenced across the RBD, the number of mutations in each RBD is shown in the histogram above. (TIF) [file ppat.1011545.s001.tif]

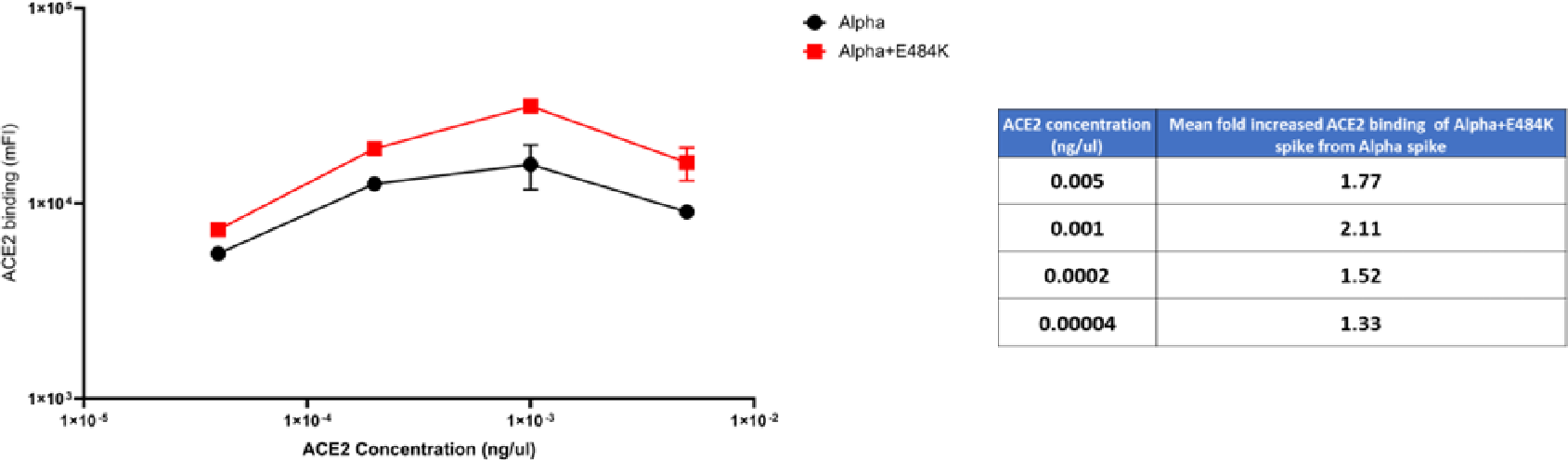

Supplement: S2 Fig — HEK-293T cells were transfected with Alpha and Alpha+E484K plasmids. 24 hours later cells were dissociated and incubated with recombinant hACE2-Fc(IgG) (Abcam ab273885) for 30 minutes. The cells were then washed and incubated with a secondary antibody against human Fc (IgG) (Abcam ab98622) for 30 minutes. ACE2 binding was measured as the median fluorescence intensity using flow cytometry. ACE2 binding was measured across a range of concentrations of recombinant hACE2-Fc(IgG) (Abcam ab273885), with all measurements being conducted in duplicate. The table shows the fold increase in ACE2 binding by Alpha+E484K relative to Alpha. (TIF) [file ppat.1011545.s002.tif]

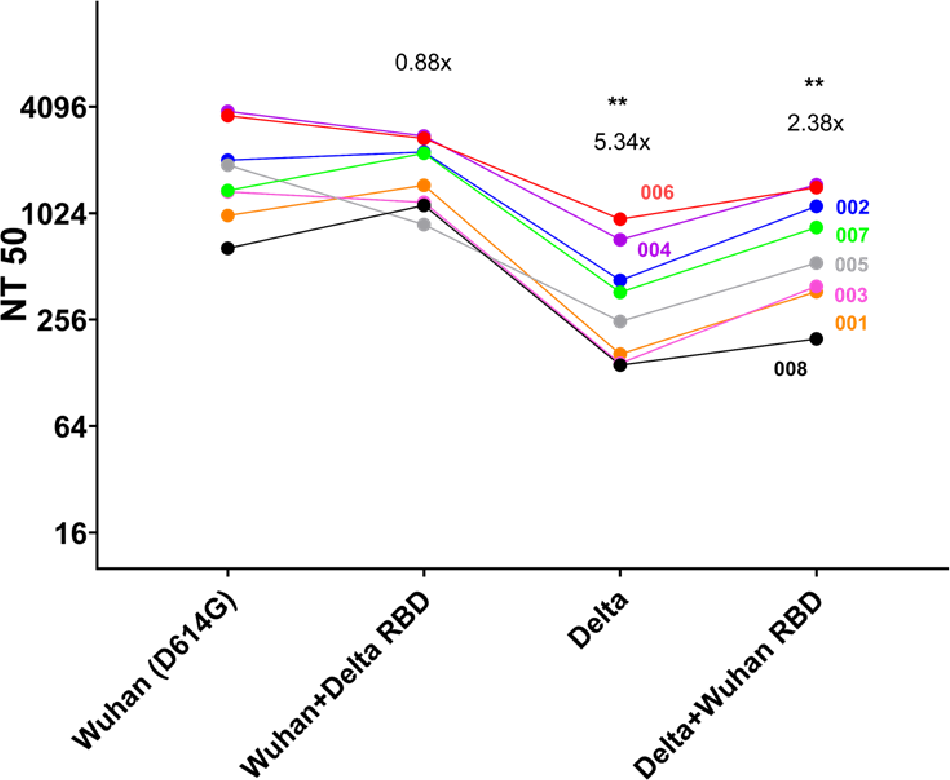

Supplement: S3 Fig — Pseudovirus neutralisation assays using double dose BNT162b2 vaccine sera against chimeric spikes involving domain swaps between Delta spike and Wuhan(D614G) spike. Shown are the median fold differences in NT50 from Wuhan(D614G) pseudovirus. HIV based pseudovirus platform with hACE2 over-expressing HEK-293T cells used for neutralisation assays. (TIF) [file ppat.1011545.s003.tif]

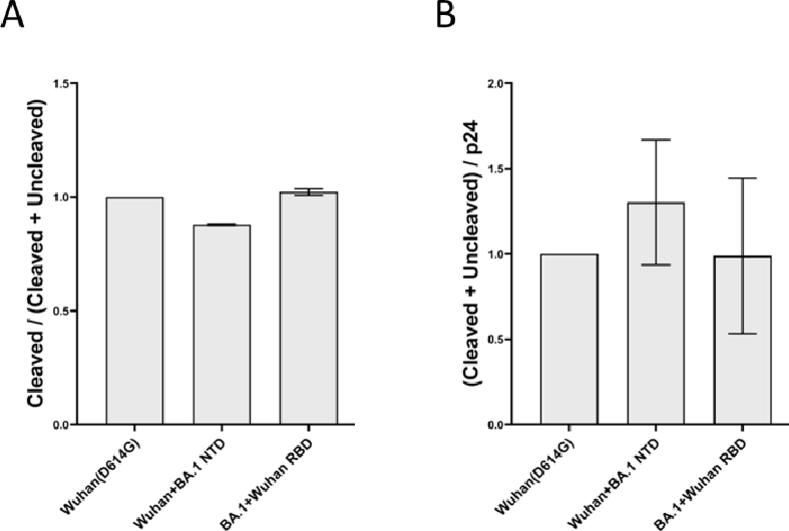

Supplement: S4 Fig — All quantification is normalised against Wuhan (D614G). (A) Proportion of cleaved to total spike. (B) Total spike per pseudovirus. Error bars represent standard error of the mean. N = 2. (TIF) [file ppat.1011545.s004.tif]

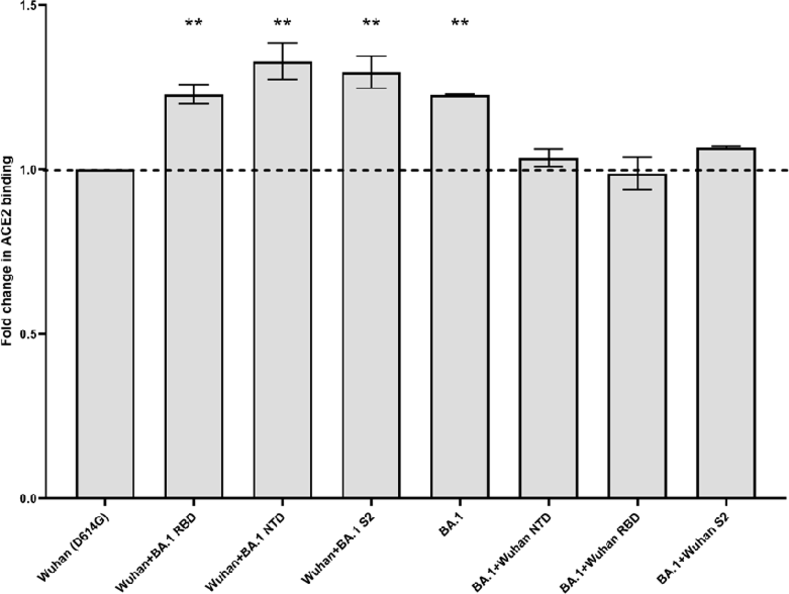

Supplement: S5 Fig — ACE2 binding was measured by flow cytometry. HEK-293T cells were transfected with the respective plasmid, 24 hours later cells were dissociated and incubated with sACE2-Fc-IgG-mScarlet for 1 hour, before measuring the median fluorescence intensity. The mfi was corrected to spike expression in the same way as described in Fig 1. Shown is the relative difference in mfi to Wuhan(D614G). n = 2. ** p value < 0.001 using one-way ANOVA relative to Wuhan(D614G). (TIF) [file ppat.1011545.s005.tif]

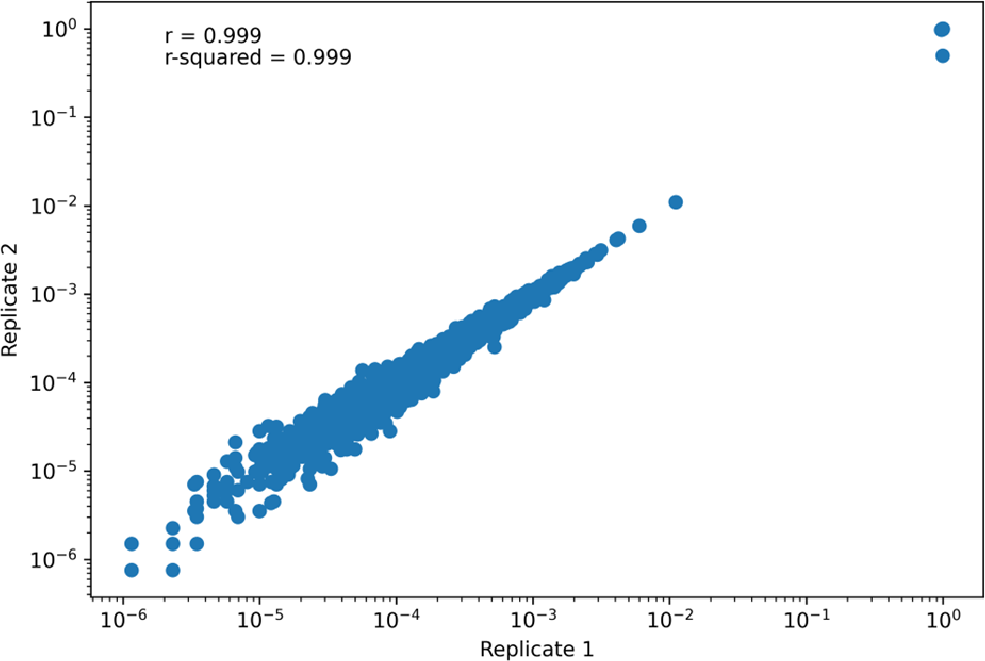

Supplement: S6 Fig — The RBD of the Alpha plasmid library was sequenced independently in duplicate by NGS. The proportion of reads containing each point mutation was calculated. The axes represent the proportion of reads for each point mutation in each of the 2 sequencing runs. Point mutations with proportions differing by a greater than 4-fold difference between replicates were excluded from the library. The scatter plot shows the correlation between proportions of point mutations included in the subsequent analyses. R value (Pearson rank coefficient). (TIF) [file ppat.1011545.s006.tif]

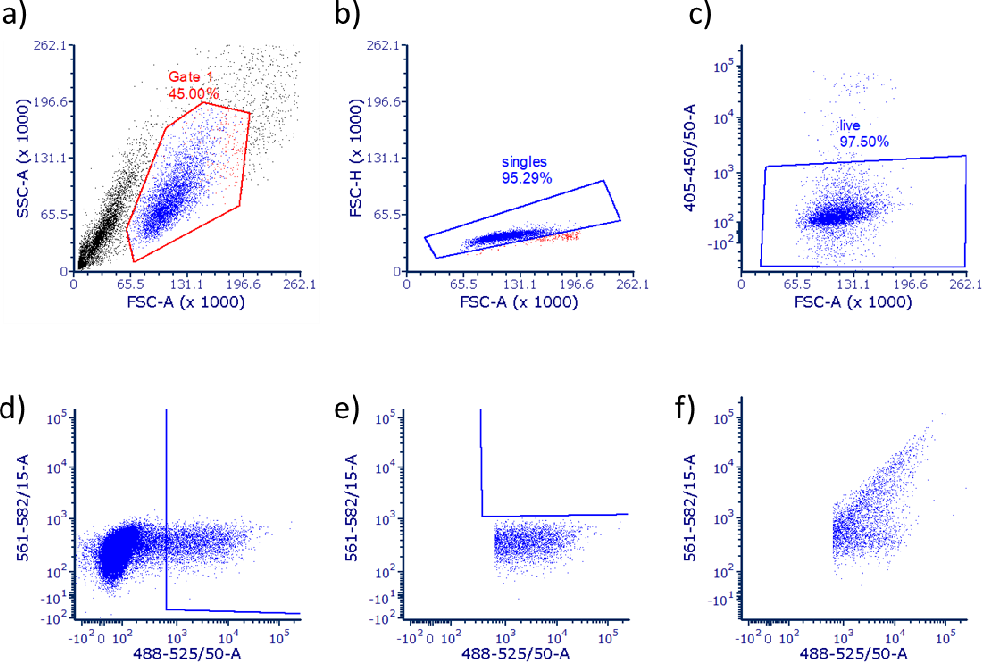

Supplement: S7 Fig — a. FSC-A against SSC-A to remove dead cells and debris. b. FSC-A against FSC-H to remove doublets. c. FSC-A against 405-450/50-A to remove dead cells by staining with DAPI. D. 488-525/50-A (spike expression) against 561-582/15 –A (ACE2) to select spike expressing cells. e. 488-525/50-A against 561-582/15-A gate for ACE2 positive spike expressing cells. f. 488-525/50-A against 561-582/15-A, the top 10% of ACE2 binding cells are sorted from an ACE2 positive, spike expressing population. The spike protein used were all tagged at the C-terminal end with mGreenLantern and the ACE2-IgG Fc tagged with mScarlet. (TIF) [file ppat.1011545.s007.tif]

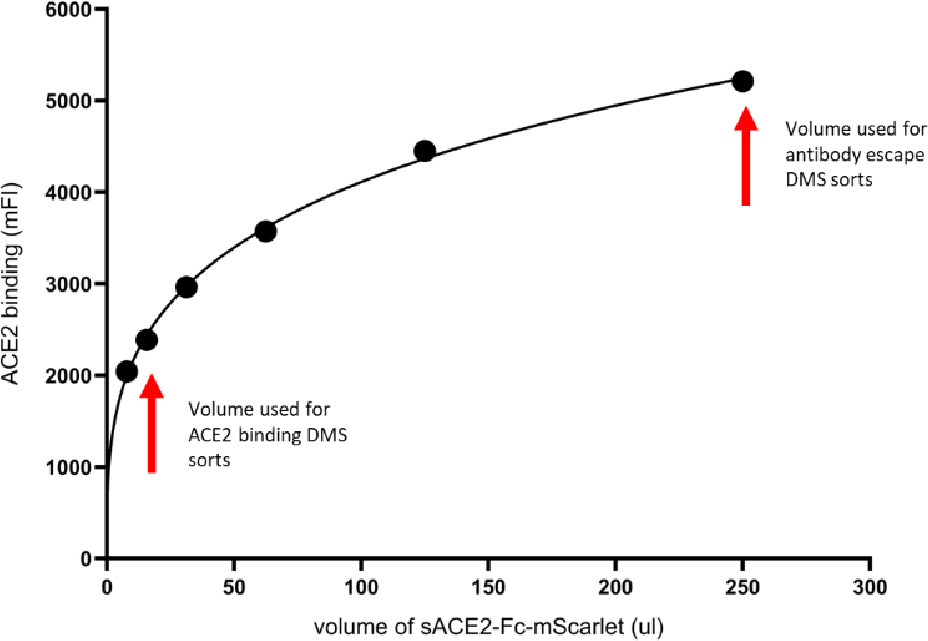

Supplement: S8 Fig — HEK-293T cells were transfected with the sACE2-Fc-mScarlet plasmid at a ratio of 1000ng per 10^6 cells. After 48 hours the supernatant was collected, filtered and aliquoted for storage at -20°C. sACE2-Fc-mScarlet from this batch was used to incubate ~5*10^5 HEK-293T cells transfected 24 hours previously with plasmid expressing Alpha SARS-CoV-2 spike tagged with mGreenLantern for 30 minutes. The Alpha SARS-CoV-2 spike tagged with mGreenLantern plasmid was transfected using 1ng of the spike expressing plasmid with 2000ng of empty plasmid per 10^6 HEK-293T cells. ACE2 binding is shown as the median fluorescence intensity (mFI) of mScarlet following incubation with a range of volumes of the sACE2-Fc-mScarlet supernatant. (TIF) [file ppat.1011545.s008.tif]
