# Supplementary material for: Deep mutagenesis scanning using whole trimeric SARS-CoV-2 spike highlights the importance of NTD-RBD interactions in determining spike phenotype
Source: PLoS Pathog. 2023 Aug 3;19(8):e1011545. doi: 10.1371/journal.ppat.1011545 (PMC10426949; doi:10.1371/journal.ppat.1011545)
Supplement: Attachment — Submitted filename: response_to_reviewers.docx [file ppat.1011545.s012.docx]

Dear Editors,

Thank you for the chance to respond to the helpful reviewers’ comments and also to your own.

We now submit a revised manuscript in which we highlight the changes we have made to our manuscript based on this feedback, we address all comments as detailed below with either new data or amended words in the text.

**From the editor:**

**In addition, I would add the following point not raised by the reviewers: the very large increase in ACE2 affinity due to E484K is surprising. Many prior studies have looked at affinity enhancing mutations, and have not generally found E484K to have a large effect even in a N501Y background. Given the surprising nature of this result, some further validation should be performed using an orthogonal assay, or at least flow cytometry at titrated levels of ACE2.**

We agree that Starr et al had seen no increase using the RBD in the yeast system. We have now performed further experiments to confirm our original findings using titrated levels of ACE2 as suggested by the editor, amended our text and included a new supplementary figure 2. We do not know why we see the increase in contrast to Starr et al. Three other studies using biolayer inferometery (PMID: 34400835) and surface plasmon resonance (PMID: 34435953 and PMID: 34023401) also find E484K increases ACE2 binding by the SARS-CoV-2 RBD in the presence and absence of N501Y. Additionally, the relative size of the increase in ACE2 binding conferred by the mutation E484K is comparable between these studies and our own.

Line 114 has been amended; “We note that E484K did not appear to increase ACE2 binding in a yeast DMS scan using an RBD containing N501Y. To confirm the increase in binding we were observing in the context of whole Alpha Spike, we measured ACE2 binding across a range of different concentrations of purified recombinant ACE2 and confirmed the ACE2 binding increase to the E484K mutation at each of these concentrations (Fig S2).”

Fig. S2. The E484K mutation increases Alpha SARS-CoV-2 spike binding to ACE2. HEK-293T cells were transfected with Alpha and Alpha+E484K plasmids. 24 hours later cells were dissociated and incubated with recombinant hACE2-Fc(IgG) (Abcam ab273885) for 30 minutes. The cells were then washed and incubated with a secondary antibody against human Fc (IgG) (Abcam ab98622) for 30 minutes. ACE2 binding was measured as the median fluorescence intensity using flow cytometry. ACE2 binding was measured across a range of concentrations of recombinant hACE2-Fc(IgG) (Abcam ab273885), with all measurements being conducted in duplicate. The table shows the fold increase in ACE2 binding by Alpha+E484K relative to Alpha.

**Part II – Major Issues: Key Experiments Required for Acceptance**
Please use this section to detail the key new experiments or modifications of existing experiments that should be absolutely required to validate study conclusions.

Generally, there should be no more than 3 such required experiments or major modifications for a "Major Revision" recommendation. If more than 3 experiments are necessary to validate the study conclusions, then you are encouraged to recommend "Reject".

**Reviewer #1: 1. Reproducibility of the DMS was not assessed. How many independent biological replicates has been done for the screening? What is the correlation between replicates? These were not mentioned throughout the manuscript.**

Technical replicates to assess DMS reproducibility were not performed. Rather we used pooled biological repeats (two or more independent cell sorts) where the cells were pooled, prior to RNA extraction, reversed transcribed, PCR amplified and sequenced using next generation sequencing. A selection of the highest scoring hits from the screen were confirmed using flow cytometry for ACE2 binding or pseudovirus neutralisation assays for antibody escape.

**2. The effect of RBD mutations on ACE2 binding have been measured by two previous DMS studies (PMID: 33597251 and PMID: 32841599). What is the correlation between the data in this present study with those two previous studies?**

Formally assessing complete published dataset correlation with this study was not undertaken for ACE2 or monoclonal antibody binding. Differences in methodology, scoring systems and inclusion/exclusion cut-offs make total dataset comparison difficult to interpret. The two studies mentioned, PMID: 33597251 and PMID: 32841599 both use a Wuhan based RBD, it has already been shown that epistasis with N501Y determines the ACE2 binding phenotype of amino acids at position 498. Instead, we now make comparisons with two other evolution studies that have a 501Y containing RBD (PMID: 35762884 and PMID: 34400835). PMID: 34400835 was a directed evolution study using error prone PCR. Although they started with a Wuhan RBD, N501Y fixed early in the rounds of selection and the subsequent mutations were added to N501Y. In our study, to reduce the impact of noise from low frequency reads we filtered our results by focussing on RBD positions that directly interact with ACE2 (PMID: 32841599).

We have now made that clearer in the text body adding at line 106 “We focused our analysis to the RBD residues that were in the interface with ACE2 receptor, and also those that are altered in Omicron BA.1 Spike.”

Our study was designed to identify the mutations having the greatest effect on increasing ACE2 binding. Using the same principle with PMID: 33597251 and PMID: 32841599, the top three performing RBD mutations that directly interact with ACE2 in PMID: 33597251 were Q493Y, Q498R and Y453F, while in PMID: 34400835 they were E484K, Q498R and N460K and in our study they were E484K, Q498R and L452R (a small increase in enrichment compared to E484K and Q498R, so was not explored further in this study). All three studies highlight that the majority of mutations do not increase ACE2 binding, however there is significant variation in the mutations that are selected out by each study, and this is likely due to the differences in methodology used as described above. Despite this, all three studies identify Q498R, while this study and PMID: 34400835 both identify E484K and Q498R as mutations having the largest effect on increasing ACE2 binding.

Zahradnik et al. (DOI: 10.1038/s41564-021-00954-4), Starr et al (DOI: 10.1126/science.abo7896), Bate et al. (DOI: 10.1371/journal.ppat.1010733)

We have also added at Line 265: “E484K was not predicted to increase ACE2 binding in a yeast DMS, however did fix early in a directed evolution study of the RBD after N501Y looking for a high affinity RBD using rounds of error-prone PCR. Biolayer interferometry measurements have confirmed the addition of E484K to a N501Y RBD does increases the affinity to ACE2. In addition, 2 independent studies using surface plasmon resonance to measure RBD binding to ACE2 also showed E484K increases ACE2 binding both in the presence and absence of N501Y. ”

**3. Figure 4 E,F,G: Neutralization assays on BA.1 should also be included as a control.**

BA.1 data is now included and shows complete escape by BA.1 from these antibodies at all concentrations used.

Amendment: Figures 4E,F,G are now Figures 5A, B, C.

**4. Page 8: “The enhancement of ACE2 binding by the BA.1-NTD was not conferred unless the BA.1-RBD was coupled with its cognate NTD (Fig S4).” This claim needs to be either modified or supported by including Wuhan spike + BA.1 RBD, Wuhan spike + BA.1 NTD, and Wuhan spike + BA.1 RBD + BA.1 NTD in the experiment, since it is possible that S2 domain also plays a role here. Currently, domain swapping in Fig S4 was only performed using BA.1 spike (i.e. BA.1 spike + Wuhan RBD and BA.1 spike + Wuhan NTD).**

Figure S5 (previous S4) has been updated to include the suggested domain swaps and text changed accordingly.

Previously stated: “Replacing the NTD of BA.1 with the Wuhan-NTD led to a 20% decrease in ACE2 binding”

Line 248 Changed to: “The BA.1 spike binds ACE2 20% better than Wuhan (D614G) spike. Replacing the RBD of BA.1 with Wuhan RBD returns ACE2 binding back to the level of Wuhan (D614G), surprisingly replacing the NTD and S2 domain of BA.1 with Wuhan equivalents reduce the ACE2 binding of spike by a similar level(Fig S5). The replacement of Wuhan spike domains with any of BA.1 NTD, RBD and S2 domains increases ACE2 binding to a similar level to BA.1 whole spike. This highlights the importance of domains outside of the RBD in determining ACE2 binding of whole spike (Fig S5).”

**Reviewer #2:**

**1. Other citations with SARS-CoV-2 DMS platforms: PMID 36417523 and Cell doi 10.1016/j.cell.2023.02.001. Both are whole-spike platforms and probably should be mentioned alongside the methodology discussed here. Another important citation and experiments to contrast results with is PMID 35988543, see point below.**

All three references mentioned have now been included.

Previously stated “However, these have been performed using just the RBD, which when expressed by itself is monomeric.”

Line 56 Changed to: “However, the majority of these studies have been performed using just the RBD, which when expressed by itself is monomeric. Whilst evolution studies using monomeric RBD have successfully predicted the emergence of key RBD mutations, the maps might be improved by using SARS-CoV-2 spike in its native trimeric form in which both intra and inter-domain epistasis can play out. Increasingly studies with deep mutagenesis using whole spike are contributing to the knowledge base of mutational effects. These have included mammalian cell displays of SARS-CoV-2 spike to explore the effect of mutations on the NTD[11] and using pseudovirus systems with whole spike deep mutational scanning (DMS) libraries[9].”


**2. It is certainly unexpected as the authors say to see that there is a greater loss in serum neutralizing potency for the Wuhan-Hu-1 spike with the BA.1 NTD versus the BA.1 RBD (and its individual antibody-escape mutations). I do believe that NTD mutations can impact RBDs, but I do think the particular findings here warrant a more careful consideration. These results differ quite dramatically from the extensive chimera experiments performed by Javanmardi et al. (PMID 35988543 reference listed above), who used a mammalian spike display platform to extensively characterize BA.1 mutations and chimeras for mAb binding and other biochemical properties. Their Wuhan-1 + BA.1 NTD or BA.2 NTD constructs (Fig. 4A) do not impact binding by any of the RBD mAbs in their panel (including overlap with mAbs seen here), though these constructs for example do exhibit enhanced spike expression (consistent with Fig. 4H). Because Javanmardi et al. conduct binding assays which are more straightforward than neutralization assays, I think these results need to be carefully considered in light of contrasting results here. In particular, this comparison makes me wonder if something indirect is happening in the relationship between spike function/expression and pseudoviral infection and/or neutralization in the current manuscript that could give rise to artefactual or nonspecific changes in neutralizing potency due to indirect spike features and not antibody escape per se. For example, results from the present manuscript and Javanmardi et al. indicate that many of these spike chimeras can differ in spike stability/expression or ACE2 binding, and therefore presumably infectivity. The current study states that neuts were performed setting each experiment to an equal infectious titer. However, if that requires adding substantially more pseudoviral particles (or more directly, more spike protein) for some constructs versus others, then if serum antibodies or mAbs are not present in sufficient molar excess, couldn’t this systematically alter neutralizing IC50s by excess spike depletion of limiting mAb? I think the authors need to consider how much spike (functional or not) is present in different preps and consider whether that is systematically associated with their neutralization effects of chimeras. Regardless of this specific hypothesis I suggest here for the discrepencies in results, I think the authors need to carefully discuss their results in relationship to the Javanmardi et al. results in the present manuscript, as this is crucial to the interpretation of their data.**

We thank the reviewers for highlighting this discrepancy in findings between the two studies. This prompted us to do further work to understand the difference in findings between the two studies. The Javanmardi et al. (PMID 35988543) study used pre-fusion stabilised spike constructs for their mammalian display. They used 6 proline substitutions in the S2 of spike to increase spike expression on the mammalian cell surface. We hypothesised that the effect we see in our study of the NTD domain altering RBD neutralisation by a RBD targeting antibody was related to the NTD altering the presentation of the RBD and making it harder to neutralise. Using 6 proline substitutions to fix spike in a pre-fusion stabilised state would likely alter the inter-domain mobility between the NTD and RBD and prevent the effect of the BA.1 NTD on the RBD being realised.

To explore this, we designed matching chimeric spike proteins with and without the 6 proline substitutions in the S2 as described in Javanmardi et al. (PMID 35988543) and measured mAb (Ly-CoV-016, REGN 10933, REGN 10987) binding to these spike constructs displayed on mammalian cells. Indeed, we confirmed our previous finding in the non-stabilised spikes that the BA.1 NTD reduced mAb binding to a Wuhan RBD compared to Wuhan whole spike. In contrast, in the pre-fusion stabilised spikes we found no difference in mAb binding to a Wuhan RBD when coupled with a BA.1 NTD, agreeing with the findings of Javanmardi et al. (PMID 35988543).

This highlights that NTD interdomain effects on the RBD are masked by pre-fusion stabilisation of spike and we suggest that findings from studies using pre-fusion stabilised spikes should take this into account. We believe this additional information provided by the new experiments enhances our manuscript by bringing some mechanistic insight to our findings.

We have updated our results with the new data, figure 5E,F,G.

And amended the text at Line 234

“These findings contrasted with the findings from Javanmardi et al. who showed little effect of BA.1 NTD domain on the binding of RBD directed mAbs. We hypothesized the different findings may be related to the use of pre-fusion stabilized SARS-CoV-2 spikes by Javanmardi et al. who engineered 6 proline substitutions in S2 to increase spike expression. The effect we see of the BA.1 NTD on RBD directed mAbs may require a native S2 to transmit a dynamic interaction between the BA.1 NTD and neighbouring RBD. To assess this hypothesis, we engineered matched chimeric spike proteins with domains from BA.1 and Wuhan spike with and without pre-fusion stabilization in S2 and compared binding of RBD-directed mAbs using flow cytometry (figure 5E, F, G). We found that pre-fusion stabilization abrogated the effect of the BA.1 NTD on mAb binding to the RBD in agreement with Javanmardi et al (figure 5E, F, G). However, in the non-stabilized spike, the BA.1 NTD significantly reduced RBD-directed mAb binding in agreement with our pseudovirus neutralization data. This implies that the interdomain effects between the NTD and RBD that impact antibody binding and likely also on ACE2 binding require the S2 to be mobile (figure 5E, F, G).”

We also amended the discussion as follows:

Line 325 “This effect of the BA.1 NTD on antibody binding of the RBD was only present with a native S2 domain and not when the spike was pre-fusion stabilised[31], suggesting that protein mobility is required to transmit the impact from one domain to the next.”

**3. Data is only available upon request. At minimum, a table of all of the deep mutational scanning enrichments should be made available as supplementary data. Raw sequencing data is typically required to be uploaded to a repository, as well. I believe PLOS policy is that raw data for all plots should be available, too.**

All the sequence data will be deposited in the Short Read Archive <https://www.ncbi.nlm.nih.gov/sra/PRJNA962104>

Line 484 added: “All raw sequencing data can be found at: https://www.ncbi.nlm.nih.gov/sra/PRJNA962104”

**Part III – Minor Issues: Editorial and Data Presentation Modifications**
Please use this section for editorial suggestions as well as relatively minor modifications of existing data that would enhance clarity.

Reviewer #1:

**1. Throughout the manuscript, “SARS-CoV2” should be changed to “SARS-CoV-2”.**

All instances of “SARS-CoV2” changed to “SARS-CoV-2”. Changes are highlighted in yellow.

**2. Deep mutational scanning of SARS-CoV-2 RBD using whole trimeric spike and mammalian display has been done and published before (PMID: 33597251). Moreover, antigenic escape screen using whole trimeric spike and mammalian display has also been done and published before (please see PMID****: 34919820). However, the authors stated that they are the first to adopt such strategies. Therefore, the innovation part of this manuscript should be toned down.**

‘We have toned down our words as requested and referenced the two other papers. We note however that PMID: 33597251was only with mAbs against the NTD and used alanine scanning not DMS’.

Line 56 “However, these have been performed using just the RBD, which when expressed by itself is monomeric”

Changed to : “However, the majority of these studies have been performed using just the RBD, which when expressed by itself is monomeric. Whilst evolution studies using monomeric RBD have successfully predicted the emergence of key RBD mutations, the maps might be improved by using SARS-CoV-2 spike in its native trimeric form in which both intra and inter-domain epistasis can play out. Increasingly studies with deep mutagenesis using whole spike are contributing to the knowledge base of mutational effects. These have included mammalian cell displays of SARS-CoV-2 spike to explore the effect of mutations on the NTD[11] and using pseudovirus systems with whole spike deep mutational scanning (DMS) libraries.”

Line 263 previously stated “Here, we developed a method for DMS using trimeric whole spike displayed on human cells.”

Changed to “Here, we used DMS with trimeric whole spike displayed on human cells.”

Line 331 previously stated “This study provides a proof of principle for the use of deep mutagenesis scanning with whole trimeric spike displayed on mammalian cells to screen for mutations that increase ACE2 binding and escape antibodies.”

Changed to “This study uses deep mutagenesis scanning with whole trimeric spike displayed on mammalian cells to screen for mutations that increase ACE2 binding and escape antibodies.”

**3. Figure 1A: What is the green star in the scheme? An antibody, or a bead coated with antibodies?**

Thanks for highlighting this, the green star was meant to represent a SARS-CoV-2 spike mutant. The figure legend has been updated to include this for greater clarity.

**4. Page 6: the meaning of “ACE2 binding normalised for spike expression” needs to be clarified. It is unclear what normalization procedure was performed here.**

Normalisation has been changed to ‘corrected for spike expression’.

Spike constructs were tagged with mGreenLantern at the C-terminal end, allowing expression of spike by cells to be measured using the signal from mGreenLantern. ACE2 binding was measured using the median fluorescence intensity signal from the ACE2-IgG(Fc)-mScarlet. ACE2 binding was measured from a population of cells with the same level of spike expression. Spike expression was gated to the lowest level of spike expression with detectable ACE2 binding and extended for half a log, while remaining on the linear portion of the ACE2 binding – spike expression plot. An example of this is shown by the green box in the dot plot in Figure 1C. By taking measurements of ACE2 binding from lower levels of spike expression, differences in ACE2 binding related to differences in spike expression can be avoided.

The methods have been updated.

Line 432 Added: “Spike constructs were tagged with mGreenLantern at the C-terminal end, allowing expression of spike by cells to be measured using the signal from mGreenLantern. ACE2 binding was measured using the median fluorescence intensity signal from the ACE2-IgG(Fc)-mScarlet. ACE2 binding was measured from a population of cells with the same level of spike expression to avoid differences in spike expression caused by a mutation affecting measurements of ACE2 binding. Spike expression was gated to the lowest level of spike expression with detectable ACE2 binding and extended for half a log, while remaining on the linear portion of the ACE2 binding vs spike expression plot. An example of this is shown by the green box in the dot plot in Figure 1C.”

**5. Figure 2: the color scale of the heat map is a bit confusing because there are two light colors at the extremes and dark colors in the middle.**

The colour scale for all heatmaps have been changed to a single colour on a continuous scale for improved clarity. A diverging scale was avoided, as our heatmaps all start from 0, with WT mutations being classed as 0 and we only aimed to identify mutations being positively selected for ACE2 binding or antibody escape.

**6. Page 7: “E3484A” should be “E484A”.
7. Page 8: “E4984A” should be “E484A”.
8. Page 8: “(Fig 4D & table S3)” should only be “(Fig 4D)”.**

Apologies, thanks for pointing out. These have all been corrected.

**9. In the methods section: “Co-transfection with an empty plasmid reduces the number of the coding plasmids that are transfected into a single cell [3, 6-8]”. I am not sure if the references are correct here because none of those studies seem to use this method.**

Apologies, error on our part. References now corrected to the following:

DOI: 10.4049/jimmunol.1800343

DOI: 10.1074/jbc.RA118.006173

DOI: 10.1126/science.abc0870

DOI: 10.1126/sciadv.abf1738

**10. In the methods section, the authors mentioned the possible shedding of S1 of spike during sorting. Did the author modify the furin cleavage site to minimize such issue?**

The furin cleavage site was not modified. The observation that the ACE2 binding signal decreases over time during the sorts was previously noted in another study (DOI: 10.1126/sciadv.abf1738). The gates set for sorting were regularly monitored and adjusted if required to maintain a fixed percentage of the highest ACE2 binding cells. We did not want to alter the furin cleavage site as we believed this may alter the inter-domain interactions that occur in unaltered SARS-CoV-2 spike.

**11. What is the rationale of using the adjusted enrichment scores when analyzing antibody escape? It is unclear what is the point of including (number of amino acids at this position with enrichment score > 2)/(number of other possible amino acids) as a factor in the calculation of adjusted enrichment scores.**

This is addressed in the response to question 12.

**12. As shown in Figure S5, some mutations have extremely low frequency. Given that the error rate of Illumina sequencing typically ranges from 0.1% to 1%, it can be difficult to distinguish between real mutations and sequencing errors. As a result, the phenotypic measurement for low frequency variants in DMS can be very noisy. Did the authors apply a frequency cutoff to filter out low frequency variants?**

We used a frequency cut-off of 0.001%, then values differing by greater than 4-fold between two independent sequencing runs of the original plasmid library were excluded to reduce the likelihood these were due to sequencing error. To further reduce the effect of noise, mutations at the ACE2 binding face of the RBD were focused on, as mutations having a positive effect on ACE2 binding would be expected to interact with ACE2. For the antibody escape screens, to further reduce noise, an adjusted enrichment score for antibody escape was used. RBD positions important in antibody escape will generally have multiple mutations at this position capable of causing escape, since important sites of antibody escape generally correlate with structural data of the points of interaction between the amino acids of the antibody and the target, it would be expected that many amino acid substitutions at this site would have a deleterious effect on antibody binding leading to escape due to the differing physicochemical properties of the substituted amino acid.

We accept the possibility of some noise in the overall results due to using a low frequency cut-off, however we have focused on only the high scoring mutations in regions of predicted relevance. Additionally, we have sought to confirm the results of our scans using flow cytometry or pseudovirus neutralisation assays. While we accept there may be mutations in our dataset whose score is attributed to noise we have not reported on any that could not be confirmed by validation in this publication or have been previously identified. For instance, in the ACE2 binding screen, we identify the mutations E484K and Q498R, which we confirm using flow cytometry and have previously been identified. In the mAb antibody screens, we identify positions of escape that correlate with structural data and have been identified in other DMS escape screens with the exception of position 477, which we confirm using pseudovirus neutralisation assays.

The following has been added to the methods:

Line 474 “A frequency cut-off of 0.001% was used in determining the proportions of variants in the library, then values differing by greater than 4-fold between two independent sequencing runs of the original plasmid library were excluded to reduce the likelihood these were due to sequencing error. To further reduce the effect of noise, mutations at the ACE2 binding face of the RBD were focused on as mutations having a positive effect on ACE2 binding would be expected to interact with ACE2. For the antibody escape screens, to reduce noise, an adjusted enrichment score for antibody escape was used. RBD positions important in antibody escape will generally have multiple mutations at this position capable of causing escape, so greater weighting was given to mutations occurring at positions with multiple other enriched mutations (see adjusted enrichment equation below).”

**13. What are the concentrations of sACE2-Fc-mScarlet and monoclonal antibodies being used in the DMS experiment?**

The sACE2-Fc-mScarlet used for the DMS screens was produced as an unconcentrated supernatant following transfection of HEK-293T cells, consequently volumes of the sACE2-Fc-mScarlet are used. sACE2-Fc-mScarlet from a single batch that had been aliquoted and frozen was used for all experiments. A titration curve of sACE2-Fc-mScarlet binding against HEK-293T cells expressing spike has been added to the supplementary data (supplementary figure 8) to show the rationale behind the choice of volumes.

sACE2-Fc-mScarlet was produced in a single batch following transfection of HEK-293T cells at a ratio of 1000ng per 10^6 cells. 48 hours later the supernatant was collected, filtered and aliquoted and stored at -20^o^C.

The sACE2-Fc-mScarlet was titrated against spike expressing cells using the same plasmid transfection strategy as for the screens to identify the volume of sACE2-Fc-mScarlet to be used in the ACE2 binding screen and for the antibody escape selection. To select for mutations that increase ACE2 binding the most, a low volume of the supernatant was chosen that was sub-saturating to more easily distinguish mutations having a beneficial effect. For this reason, a volume of 31.25ul per 5 x 10^5 cells was chosen (supplementary figure 8).

In contrast to the screen for mutations that increase ACE2 binding, for the antibody escape screens a volume of the sACE2-Fc-mScarlet supernatant was chosen that would be expected to bind all spike expressing cells in the absence of neutralisation, thus 250ul per 5X10^5 cells was chosen, as this was close to a saturating volume of sACE2-Fc-mScarlet (supplementary figure 8).

To select for antibody escape mutations, a concentration of monoclonal antibody was chosen that reduced the ACE2 binding signal of a population of cells expressing the spike library by approximately 50%. Ly-CoV-016 (400 ng/mL), REGN 10933 (80 ng/mL, REGN 10987 (160 ng/mL).

The methods have been updated with this information.

Line 396 “For the antibody escape sorts a concentration of mAb or dilution of sera was chosen following titration with the Alpha spike RBD library that reduced ACE2 binding of the total population of cells by approximately 50%. Alpha spike expressing HEK-293T cells were initially incubated with the mAb or sera for 30 minutes, followed by washing with FACS buffer and then another incubation with a saturating volume of sACE2-Fc-mScarlet supernatant (supplementary figure 8). The concentrations of mAbs used for each sort are as follows: Ly-CoV-016 (400 ng/mL), REGN 10933 (80 ng/mL, REGN 10987 (160 ng/mL), while the dilutions of each sera used can be found in table S1.”

**14. In the methods section, the authors mentioned that “A target of ~10,000 cells were aimed to be sorted for each selection”. This is problematic considering the library size is more than 4,000. Sorting such low numbers of cells would not be sufficient to resolve the affinity hierarchy of all mutants. The authors have likely sorted a lot more cells than 10,000, but it was not mentioned in the manuscript and should be clearly stated.**

Batches of 22x10^6 HEK-293T cells were sorted for each screen. 11x10^6 HEK-293T cells were stained, incubated and washed at a time. Approximately 5-10% of these cells expressed SARS-CoV2 spike due to the method of transfection used. This means 5x10^5 – 10^6 HEK-293T cells express the spike library. For a library size of approximately 4200, this means each variant in the library is represented at least 119 times (5x10^5 / 4200) assuming each variant is equally represented. From this total spike expressing cell population, the top 10% of ACE2 binding spike expressing HEK-293T cells were sorted until at least 10,000 cells were collected. If less than 10,000 target cells were collected in a single sort, further sorts were then conducted until 10,000 target cells were collected. The target population of cells were frozen at -80^o^C and pooled with cells from the subsequent sort if required.

The methods have been updated with this further detail:

Added text:

Line 404 “Batches of 11x10^6 HEK-293T cells were stained, incubated, and washed at a time. Approximately 5-10% of these cells expressed the SARS-CoV-2 spike library due to the method of transfection used. From this total spike expressing cell population, the top 10% of ACE2 binding spike expressing HEK-293T cells were sorted until at least 10,000 cells were collected. If less than 10,000 target cells were collected in a single sort, further sorts were then conducted until 10,000 target cells were collected. The target population of cells were frozen at -80oC and pooled with cells from a subsequent sort if required.”

**15. Fig S5 and S6 are not cited in the main text.**

Line 378 S6 cited in methods – in the methods -RBD library construction

Line 390 S7 cited in methods – FACS sorting.

**16. The authors should deposit their raw sequencing data to a public database (e.g. Sequence Read Archive).**

All the sequence data will be deposited in the Short Read Archive <https://www.ncbi.nlm.nih.gov/sra/PRJNA962104>

**Reviewer #2:**

**Because neutralization sensitivities to different classes of mAbs can differ depending on cell line and ACE2 expression levels (Farrell et al. Viruses 2022, Lempp et al. Nature 2021), it would be helpful in the figure legends on neutralization assays to state the pseudovirus platform and cell lines used.**

The following line has been addended to all figures featuring pseudovirus neutralisation assays:

“HIV based pseudovirus platform with hACE2 over-expressing HEK-293T cells used for neutralisation assays.”
