# Supplementary material for: Deep mutagenesis scanning using whole trimeric SARS-CoV-2 spike highlights the importance of NTD-RBD interactions in determining spike phenotype
Source: PLoS Pathog. 2023 Aug 3;19(8):e1011545. doi: 10.1371/journal.ppat.1011545 (PMC10426949; doi:10.1371/journal.ppat.1011545)
Supplement: Attachment — Submitted filename: Responses to reviewers comments.docx [file ppat.1011545.s013.docx]

Dear Editors,

Thank you for the chance to respond to the helpful reviewers’ comments and also to your own.

We now submit a further revised manuscript in which we highlight the changes we have made to our manuscript based on this feedback, we address all comments as detailed below with either new data or amended words in the text.

**- Reviewer 2 highlighted in the first round of reviews how a supplemental file with all of the measurements in CSV or Excel format was not available, and it appears that such a file is still not available. The measured effects of the mutations is the primary output of this experiment, and so a file with those direct numbers needs to be included with the paper. I will reject any further revisions that do not provide such a file.**

Now uploaded as a supplementary file.

Line 503 added: “The raw enrichment scores can be found in the supplementary excel file: “Summary_enrichment_files_.xlsx”.”

**- Reviewer 1 highlights a number of points about noise, lack of replicates, etc that seem valid. I concur with the reviewer that it is not necessary to fix all of these with new experiments at this point, but any further revision should clearly state and discuss these limitations so they are transparently described.**

A paragraph on the limitations of the study has now been added to the discussion.

Line 331 added : “This study has limitations. The DMS screens were performed with only a single biological replicate and the low frequency cut-off used in analysis of the sequencing data introduces noise into the data, particularly for the variants present at low frequencies in the library. Additionally for these low frequency variants, possible enrichment may be missed due to the limits of detection of the sequencing methodology. However, we have validated the results of the most enriched mutants, to eliminate them being false positives, and accept the presence of other enriched mutations in the dataset that have not been validated could be the result of noise. In addition, it is possible that differing levels of spike expression of different spike mutants or chimeras might impact the ACE2 binding measurements we report. We attempted to mitigate against this by measuring ACE2 binding over a window of unsaturated spike expression covering a dynamic range, which was monitored using the mGreenlantern tag on Spike protein. We did not find a large difference in Spike expression that could explain the differences in ACE2 binding we report.”

- Please also pay careful attention to the other reviewer comments.

Reviewer Comments (if any, and for reference):

Reviewer's Responses to Questions

**Part I - Summary**
Please use this section to discuss strengths/weaknesses of study, novelty/significance, general execution and scholarship.

Reviewer #1: The authors have addressed most my previous comments. However, several concerns remain to be fully addressed, including major ones.

Reviewer #2: provided in initial review

**Part II – Major Issues: Key Experiments Required for Acceptance**
Please use this section to detail the key new experiments or modifications of existing experiments that should be absolutely required to validate study conclusions.

Generally, there should be no more than 3 such required experiments or major modifications for a "Major Revision" recommendation. If more than 3 experiments are necessary to validate the study conclusions, then you are encouraged to recommend "Reject".

**Reviewer #1: 1. Pooling cells from independent cell sorts does not count as a biological replicate of the DMS. Without biological replicates of the DMS, the reproducibility of the DMS cannot be accessed This is a huge concern given that the data is likely to be very noisy (see major comments #3 and #4 below). While performing another biological replicates of the DMS will likely be very time consuming, the authors should at least acknowledge the caveat of having only one biological replicate of the DMS.**

Thank you for highlighting this, we have now acknowledged this in a specific paragraph in the discussion covering the limitations of this study.

Line 331 added : “This study has limitations. The DMS screens were performed with only a single biological replicate and the low frequency cut-off used in analysis of the sequencing data introduces noise into the data, particularly for the variants present at low frequencies in the library. Additionally for these low frequency variants, possible enrichment may be missed due to the limits of detection of the sequencing methodology. However, we have validated the results of the most enriched mutants, to eliminate them being false positives, and accept the presence of other enriched mutations in the dataset that have not been validated could be the result of noise. In addition, it is possible that differing levels of spike expression of different spike mutants or chimeras might impact the ACE2 binding measurements we report. We attempted to mitigate against this by measuring ACE2 binding over a window of unsaturated spike expression covering a dynamic range, which was monitored using the mGreenlantern tag on Spike protein. We did not find a large difference in Spike expression that could explain the differences in ACE2 binding we report.”

**2. Line 248: “The BA.1 spike binds ACE2 20% better than Wuhan (D614G) spike. Replacing the RBD of BA.1 with Wuhan RBD returns ACE2 binding back to the level of Wuhan (D614G), surprisingly replacing the NTD and S2 domain of BA.1 with Wuhan equivalents reduce the ACE2 binding of spike by a similar level (Fig S5). The replacement of Wuhan spike domains with any of BA.1 NTD, RBD and S2 domains increases ACE2 binding to a similar level to BA.1 whole spike. This highlights the importance of domains outside of the RBD in determining ACE2 binding of whole spike (Fig S5).” It is possible that the domain swapping between the Wuhan (D614G) and the BA.1 spike leads to change in expression level of the spike chimera on the cell surface, therefore leading to the variation in ACE2 binding. Therefore, it is necessary to measure the spike expression level of these chimera constructs as a control.**

Spike expression was measured using the mGreenLantern tag at the C-terminal end of all spike constructs used in flow cytometry. Measurements of ACE2 binding were corrected for spike expression by measuring ACE2 binding over the same fixed level of spike expression as described in the methods. The possible limitations of this method have now been discussed in a paragraph on limitations in the discussion (see response to 1).

**3. Line 404: “Batches of 11x10^6 HEK-293T cells were stained, incubated, and washed at a time. Approximately 5-10% of these cells expressed the SARS-CoV-2 spike library due to the method of transfection used. From this total spike expressing cell population, the top 10% of ACE2 binding spike expressing HEK-293T cells were sorted until at least 10,000 cells were collected. If less than 10,000 target cells were collected in a single sort, further sorts were then conducted until 10,000 target cells were collected. The target population of cells were frozen at -80°C and pooled with cells from a subsequent sort if required.” This part is still quite confusing. It is true that as the authors stated, the transfection would generate enough cells to represent the whole library. However, if only 10,000 cells total within top 10% of the ACE2 binding spike expressing cells were collected, the results are likely to be very noisy given that the library contains >4,000 mutants.**

While the whole plasmid library contains over 4000 mutants, which are represented by at least 5*10^5 spike expressing cells from a total of 11*10^6 cells transfected cells, the sorted 10,000 cells will contain only a fraction of the mutants in the original plasmid library. The sorted 10,000 cells will contain predominantly mutants conferring the beneficial phenotype being selected for in that particular screen. From such a diverse library only a handful of mutations are positively selected for, such as seen in ACE2 binding and with the mAb escape screens where mutations only at a few positions are associated with increased ACE2 binding or escape.

Line added 420: “The whole plasmid library contains over 4000 mutants, which are represented by at least 5*10^5 spike expressing cells from a total of 11*10^6 cells transfected cells, the sorted 10,000 cells will contain only a fraction of the mutants in the original plasmid library. The sorted 10,000 cells will contain predominantly mutants conferring the beneficial phenotype being selected for in that particular screen. From such a diverse library only a handful of mutations will be positively selected for and be represented multiple times over in the sorted population of at least 10,000 cells.”

**4. Relatedly, lines 474: “A frequency cut-off of 0.001% was used in determining the proportions of variants in the library.” If a mutation has frequency of 0.001% (i.e. 1 in 100,000) and is enriched 5 times during selection, its frequency after selection will be 0.005% (1 in 20,000). However, such enrichment will unlikely be detected since only 10,000 cells were collected. Instead, there is a high chance such mutation will be absence in the collected cells and assigned an enrichment score of 0.**

Thank you for highlighting, this is now addressed in a paragraph on limitations in the discussion (see response to 1).

**Reviewer #2: I appreciate the new discussion of the current results versus Javanmardi et al. added in revision. The newly added comparison of the NTD chimera results with or without prefusion stabilization is intriguing. This seems to be consistent with some of the observations on variant spike dynamics seen in e.g. Calvaresi et al. PMID 36918534. I think this and some of the other differences between the current data and prior studies are still not always 100% clear (biologically that is, not in the writing itself), but the present study discusses these differences and adds important new data to the overall understanding of spike dynamics and emergent quaternary features.**

 **Line 198: “To test their importance, we created a BA.1 spike protein that retained these two mutations…” – I think the description of this would be better if it included in the description explicitly that this is a chimeric BA.1 spike where rest of the RBD besides these three substitutions is Wuhan-Hu-1 (if I understand correctly)**

Line 197: changed to “To test their importance, we created a BA.1 chimeric spike protein with a Wuhan RBD containing E484A, Q493R and N501Y”


**Line 114 and 264:
The cited yeast deep mutational scanning measurements for E484K report a consistent but small affinity-enhancing delta-log10Ka of between 0.11 and 0.24 in the reported backgrounds, corresponding to a 1.3- to 1.7-fold affinity increase, different than the no effect ascribed in the manuscript in these sections and in the reviewer response.

It is also incorrect in the reviewer response to discuss how the “relative size of the increase in ACE2 binding” as determined in the current assay quantitatively relates to the SPR/BLI measured affinities. The measurement here is very different – labeling at a single concentration with a dimeric ACE2 – from a true biochemical Kd as measured by BLI or SPR. Looking at the three cited studies, E484K has no effect on the Wuhan background in Zahradnik et al. in figure 2c (yeast display titration), and in Table 2 (BLI) there is no precise test of the affinity effect of E484K alone (it is simply tested in many sporadic combinatorial mutants, so its isolated effect is not clear). Barton et al. precisely find that E484K has a 1.19-fold enhancement affinity in Wuhan and 1.48-fold in N501Y background. Laffeber et al. measure E484K as having 1.4-fold affinity-enhancement in Wuhan RBD, and 1.7-fold on the N501Y background. Therefore, the BLI and SPR experiments cited, like the yeast DMS, ascribe a very small affinity-enhancing effect to E484K, at least in isolated RBD out of the spike context. It is entirely unclear how the “relative size” of increase identified here compares to these other studies because there is no absolute quantitative meaning to the deep mutational scanning enrichment or cell-surface expression labeling measurements as reported here. The only other reference is that here it is found to be on a similar order of magnitude with Q498R, whereas in various other studies Q498R (on top of N501Y) is seen to have a much larger effect on affinity than the <2-fold changes evident for E484K.

Zahradník, J., et al., SARS-CoV-2 variant prediction and antiviral drug design are enabled by RBD in vitro evolution. Nature Microbiology, 2021. 6(9): p. 1188-1198.

Barton, M.I., et al., Effects of common mutations in the SARS-CoV-2 Spike RBD and its ligand, the human ACE2 receptor on binding affinity and kinetics. eLife, 2021. 10: p. e70658.

Laffeber, C., et al., Experimental Evidence for Enhanced Receptor Binding by Rapidly Spreading SARS-CoV-2 Variants. Journal of Molecular Biology, 2021. 433(15): p. 167058.**

Line 114 changed to “We note that E484K did not appear to have as large an effect on ACE2 binding in a yeast DMS scan using an RBD containing N501Y”

Line 257 changed to “E484K was predicted to cause a small increase in ACE2 binding in a yeast DMS[29], and fixed early in a directed evolution study of the RBD after N501Y looking for a high affinity RBD using rounds of error-prone PCR[30].”

We accept the reviewer’s comments regarding the inability to make comparisons on the magnitude of ACE2 binding between our study and others due to the differing methodologies. We would re-phrase to say the aforementioned studies show evidence the addition of E484K to N501Y has independently been shown to increase ACE2 binding. The magnitude of the enrichment in our study differs from that seen in yeast DMS studies, which likely reflects the differing methodologies used.

**Data availability: I would think a supplemental table csv file listing the final enrichment values from the deep mutational scanning data analysis would be a key inclusion in the manuscript**

Supplemental tables with the enrichment values are now included.

**Part III – Minor Issues: Editorial and Data Presentation Modifications**
Please use this section for editorial suggestions as well as relatively minor modifications of existing data that would enhance clarity.

**Reviewer #1: 1. “Spike expression was gated to the lowest level of spike expression with detectable ACE2 binding and extended for half a log, while remaining on the linear portion of the ACE2 binding – spike expression plot.” I am still not entirely sure whether this procedure is well justified to correct for spike expression when measuring ACE2 binding. Half log (3-fold) difference in expression difference is still relatively large. It may be best to include a statement in the manuscript to acknowledge this caveat.**

This is now addressed explicitly in a paragraph on the limitations of this study in the discussion (see response to 1).

Reviewer #2: (No Response)
